# Effect of *Enterococcus hirae* GS22 Fermentation-Assisted Extraction on the Physicochemical and Bioactivities of Sea Cucumber Intestinal Polysaccharides

**DOI:** 10.3390/molecules29235800

**Published:** 2024-12-08

**Authors:** Xiqian Tan, Xiaoqing Wang, Fangchao Cui, Ali Zeshan, Dangfeng Wang, Xuepeng Li, Jianrong Li

**Affiliations:** 1 College of Food Science and Engineering, Tianjin University of Science and Technology, Tianjin 300457, China; 2 College of Food Science and Engineering, Bohai University, Jinzhou 121013, Chinalixuepeng@bhu.edu.cn (X.L.)

**Keywords:** sea cucumber intestine, polysaccharides, fermentation, prebiotic ability, *Enterococcus hirae*

## Abstract

The sea cucumber intestine (SI), a secondary product from sea cucumber processing, contains polysaccharides as one of its active ingredients, and fermentation is an effective method for extracting bioactive substances from food by-products. In this study, to explore the effect of *Enterococcus hirae* GS22 fermentation on the extraction of SI polysaccharides, the polysaccharides were extracted through the SI with and without *Enterococcus hirae* GS22 fermentation, and the obtained polysaccharides were designated as SC-PF and SC-P. The extraction yield, the structural characteristics, and the biological functions of the polysaccharides were then evaluated. The results indicated that *Enterococcus hirae* GS22 could grow well using SI as the substrate and that fermentation could improve the extraction yield of the polysaccharide from 0.48% to 0.63%, decrease the molecular weight (Mw), and change the monosaccharide composition. The diameter of SC-PF was smaller than SC-P, and the absolute value of the zeta potential of SC-PF was found to be lower than SC-P. Fermentation does not change the functional group or the thermal ability of the polysaccharide. SC-PF had better antioxidant ability than SC-P; the DPPH and superoxide anion scavenging ability were 96.3% and 36.5%, respectively. SC-PF also showed nearly 1.3- and 1.1-fold higher inhibition of α-glucosidase and α-amylase as compared to SC-P. The current results showed that *E. hirae* GS22 fermentation has the potential to extract SI polysaccharides with better prebiotic abilities.

## 1. Introduction

Sea cucumber is a delicious seafood containing bioactive substances such as peptides, polysaccharides, and saponins, and it has therapeutic functions that might benefit humans [1,2]. Polysaccharides from sea cucumbers are among the most important functional components and show a range of valuable properties, including hypoglycemic, antioxidant, hypolipidemic, and anticoagulant activities. Different parts of sea cucumbers, including the body wall, viscera, intestine, and ovum, all contain polysaccharides. However, most extensive research has focused on polysaccharides obtained from the body wall, while limited attention has been given to those derived from the intestine of sea cucumbers [3,4,5,6].

The sea cucumber intestine (SI) is a by-product of industrial processing, often discarded, which not only causes environmental pollution but is also a waste of food sources. It is urgent for the SI to be thoroughly utilized as a material with added value. Recent research has examined the functional characteristics of the SI and its extracts, especially SI-derived peptides, including their ability to alleviate exercised-induced fatigue [7] or enhance bone regeneration [8]. Moreover, recently, there has been increasing attention on the functions of polysaccharides from SIs. It has been proven that a sulfated polysaccharide derived from sea cucumber viscera has immune-enhancing properties [9] and anti-inflammatory properties [10]. However, further relevant research still needs to be facilitated.

The functions of most polysaccharides are relevant to their unique structures, and their functions may be affected by the extraction methods [11]. Various techniques have been used to extract useful polysaccharides from diverse food matrices [12]. Enzyme hydrolysis by different proteases has long been used to extract practical ingredients from food matrices, such as antioxidant hydrolysates obtained from soybean protein [13], antioxidant and UV-B protective hydrolysates from sea cucumber body walls [14], and antioxidant and pancreatic lipase inhibitory hydrolysates from the *Cucumaria frondose* intestine and ovum [15]. According to the current research, sea cucumber polysaccharides are held tightly by the sea cucumber body wall protein; however, they may be liberated from the body wall through protein hydrolysates [16,17]. Papain (EC 3.4.22.2) has been extensively utilized to produce hydrolysates derived from the body wall of sea cucumbers. It has been proven that it can produce functional peptides or substances from sea cucumbers and help release polysaccharides. Hence, papain hydrolysis could be an auxiliary method of extracting polysaccharides from SIs.

Apart from enzyme hydrolysis, fermentation is another practical and economical way to hydrolysate a food matrix to acquire functional compounds, which has been proven to improve the extraction yields, modify the structures, and alter the functional properties of polysaccharides from different food matrices under certain conditions [18,19], which might be due to microorganisms and their metabolites in the fermentation process, especially under the effects of diverse enzymes (protease, chitinase, and chitosanase) that could facilitate the release of bioactive substances from the food matrices. *Lactobacillus fermentum*, *Bacillus subtilis*, *Saccharomyces cerevisiae*, and *Bacillus subtilis Natto* have been used to ferment longan, wheat bran, and sea cucumber to obtain diverse kinds of polysaccharides with different functions [19,20,21]. *Enterococcus hirae* has been regarded as a potential probiotic, which has been reported to have antibacterial, anti-inflammatory [20], and cholesterol-lowering properties [21]; it also has great fermentation characteristics and can be used as the starter culture in the fermentation of animal or plant-originated food matrices, such as manufacturing goat’s milk with beneficial effects on dogs [22] and fermenting beet molasses to improve the production of lactic acid [23]. However, as far as we know, the effects of *E. hirae* on fermented sea cucumber intestines still require further research.

In this article, *E. hirae* GS22 fermentation was implemented to extract polysaccharides from SIs and aimed to investigate whether the fermentation could modify the molecular weight (Mw), chemical composition, monosaccharide composition, functional groups, and bioactivities of SI polysaccharides to investigate the potential of SI polysaccharides to be used as functional ingredients in food.

## 2. Results and Discussion

### 2.1. Bacterial Growth and pH Variation During Fermentation

**Figure 1:**
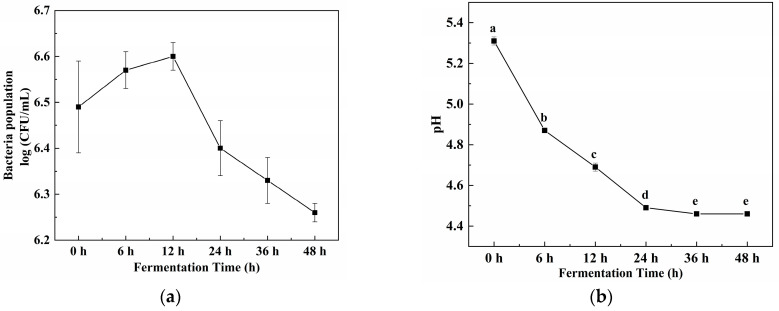
Bacterial growth and pH variation during fermentation: (**a**) bacterial count; (**b**) pH value. Different lowercase letters indicate statistically significant differences (*p* < 0.05).

The changes in bacterial count and pH throughout the SI fermentation process are illustrated in Figure 1a. The bacterial counts increased from 6.49 to 6.60 log CFU/mL over the 12 h fermentation process, after which the bacterial counts decreased gradually. However, the counts were still at 6.26 log CFU/mL until the end of the fermentation. These findings propose that *E. hirae* GS22 could survive in the SI medium. The metabolism of *E. hirae* GS22 might result in organic acid production, resulting in a decrease in pH; hence, the pH significantly dropped from 5.31 to 4.49.

### 2.2. Extraction Yield and Chemical Compositions of the Polysaccharides

Table 1 illustrates the extraction yields and physicochemical characteristics of the polysaccharides. The results showed that the fermentation significantly improved the SI polysaccharide extraction yield by nearly 1.31-fold (*p* < 0.05); the carbohydrate content, protein, reducing sugar, and uronic acid in SC-PF were also 13.40, 20.61, 39.73, and 26.09% higher than SC-P, respectively. The improvement of the polysaccharide extraction might be due to the fermentation effectively destroying the cell membrane and the bond between the polysaccharide and the protein, and it was consistent with the literature reports [24]. Compared to different methods, fermentation is a more economical way to improve the extraction yield of the polysaccharides and might endow them with excellent functional abilities [25]. Although some studies have indicated that the growth of fermentation strains utilizes sugars during the fermentation process, resulting in a reduced extraction rate and total sugar content, this study found that the polysaccharides and total sugar content extracted through fermentation were still significantly higher than those in the non-fermented group [26].

Mw is a crucial physicochemical parameter closely associated with the polysaccharides’ biological activity. In the present study, the Mw of SC-PF was less than that of SC-P, which might be due to the metabolism of *E. hirae* GS22. Previous studies suggest that polysaccharides with lower Mw generally show enhanced biological activity, such as better antitumor effects, due to the low Mw polysaccharides that could enter the tumor cells more easily [27].

Both SC-P and SC-PF are heteropolysaccharides. SC-P consists of eight different types of monosaccharides, Man, Rib, GlcA, GalA, Glc, Gal, Ara, and Fuc, with molar ratios of 2.96:7.80:0.10:0.02:7.63:1.49:0.19:1.26. Rib and Glc are the most abundant, while GalA is the least abundant. The appearance of the Ara in SC-P might originate from some of the exopolysaccharides (EPS) of Bacillus [28]. Research has found that there are certain Bacillus present in the sea cucumber intestines [29], and the sterilization method we employed cannot completely eliminate the Bacillus spores in the sea cucumber intestines. SC-PF consists of nine types of monosaccharides, Man, Rib, GlcA, GalA, Glc, Gal, Xyl, Ara, and Fuc, with molar ratios of 5.55:10.07:1.17:0.06:13.4:3.12:0.04:0.37:1.72. Glc and Rib are the two most abundant, while Xyl is the least abundant. GlcA and GalA in both SC-P and SC-PF indicate that they are acidic polysaccharides. A comparison between SC-P and SC-PF shows changes in the monosaccharide compositions after fermentation. SC-PF contains Xyl, which is absent in SC-P, which might relate to the EPSs produced by *E. hirae* 22, as some research has already reported that *E. hirae* can produce different functional EPSs [30]; however, the metabolism of *E. hirae* 22 in fermented SIs still needs to be explored. The molar ratios of monosaccharide compositions in SC-PF are slightly higher than those in SC-P, indicating that bacterial fermentation has an influence on the monosaccharide compositions and molar ratios of SI polysaccharides. This effect may be attributed to the fermentation process causing the hydrolytic cleavage of polysaccharide chains, which disrupts intermolecular hydrogen bonds and further alters the structure of the monosaccharides. Based on another study, polysaccharides rich in uronic acid content may demonstrate potent scavenging abilities and antioxidant activities [31]. The GalA contents of SC-PF (1.17%) were found to be higher than SC-P (0.1%), which suggests that SC-PF might have better antioxidant activities.

Particle size distribution is a vital index for evaluating the aggregation tendencies of polysaccharides. As shown in Table 1, the diameter of SC-PF was smaller than SC-P. The average particle sizes of SC-P and SC-PF were 2005.09 ± 82.73 nm and 1285.77 ± 168.85 nm, respectively.

Zeta potential is a characteristic that describes the distribution and orientation of surface charge, which in turn influences the overall stability of the solution. The zeta potentials of the two polysaccharides were negative, being −16.49 ± 0.53 mV and −21.42 ± 1.38 mV, respectively. The stability of a solution is positively associated with the magnitude of the zeta potential. Typically, a higher zeta potential absolute value in a dispersion system leads to increased electrostatic repulsion between molecules, resulting in enhanced dispersion and stability within solutions. The absolute zeta potential of SC-P was lower than that of SC-PF, indicating that SC-P solutions are more prone to flocculation. Moreover, the negative charge indicates that SC-P and SC-PF can provide electrons due to the presence of anions in the polysaccharide chains. The SC-PF group showed a higher absolute zeta potential value compared to the SC-P group, potentially attributed to differences in uronic acid content, as uronic acids have carboxyl groups that undergo protonation. The variation in the zeta potential and particle size of polysaccharide solutions reflects the stability of the solution [32]. The smaller particle size and larger zeta potential value of SC-PF indicate better solubility and stability than the SC-P group.

### 2.3. The Spectra and Morphology Analysis of the Polysaccharides

**Figure 2:**
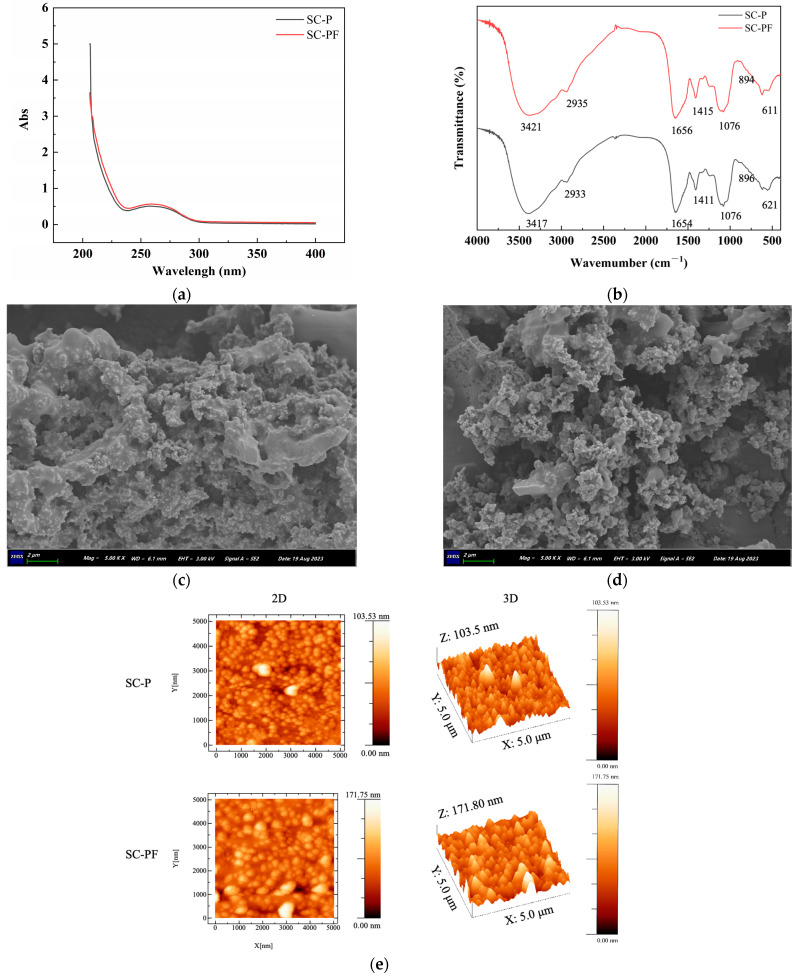
The spectra and morphology analysis results of the polysaccharides: (**a**) UV spectra; (**b**) FTIR spectra; (**c**) SEM image of SC-P (5000×); (**d**) SEM image of SC-PF (5000×); (**e**) AFM image.

Figure 2 indicates the spectra and morphology analysis results of the polysaccharides. In Figure 2a, the UV–vis spectrum displays a small absorption peak at 260 nm, which may be attributed to a certain amount of ovum being mixed with the intestinal content. The absorption spectrum showed no distinct peak at 280 nm, indicating that most of the proteins were removed during the purification process, which is in accordance with the protein content in Table 1.

The FT-IR spectra (Figure 2b) of SC-P and SC-PF show absorption bands near 3421, 2935, 1656, 1415, 1076, and 611 cm⁻^1^. The absorption peak near 3421 cm⁻¹ represents stretching vibrations of O-H bonds [33], whereas the peak around 2935 cm⁻¹ indicates stretching vibrations of C-H bonds in the methyl groups of fucoidan [34], and these peaks are characteristic of polysaccharides. The absorption bands around 1656 cm⁻^1^ and 1076 cm⁻^1^ are attributed to the stretching vibrations of C=O and C-O-C, respectively [35], indicating that both polysaccharides contain uronic acids. The signal detected at 1415 cm⁻¹ corresponds to the stretching vibration of C-H-O bonds, while the absorption peak at 894 cm⁻¹ indicates symmetric stretching of C-O-S bonds. These features indicate the presence of *β*-glucosidic linkages in both SC-P and SC-PF [36], and the band around 611 cm⁻^1^ might be related to the stretching vibration of the S-O bond, indicating that both polysaccharides are sulfated. The FT-IR spectra of SC-P and SC-PF were nearly indistinguishable, suggesting that fermentation did not change the core structural features of the SI polysaccharide.

SEM is widely used to observe the micro-morphology of polysaccharide substances [37]. The surface morphology of the polysaccharides magnified 5000 times using SEM is shown in Figure 2c. The results indicate that SC-P and SC-PF have similar morphological structures, both displaying a smooth mesh structure composed of numerous spherical particles. However, the particle size of SC-PF seems smaller than SC-P, and the porosity levels of the surface of SC-PF were a little higher than SC-P. This could be due to the metabolic activity of the strains, which may affect the structure of the polysaccharides.

AFM is an effective technique for evaluating the spatial structure and surface morphology of biomacromolecules, which has the ability to directly visualize morphological features, molecular movements, linear arrangements, clusters, and helical structures. The AFM approach has already been used to analyze the nanostructure of polysaccharides. The occurrence of luminous regions in the 2D images may be associated with the aggregation of polymers during the preparation procedure and the strong intermolecular bonds between polysaccharide molecules. Our results show that the molecular chains of SC-P and SC-PF have non-uniform structural dimensions and morphologies. The surfaces have cone-like and block-like structures, with mean heights between 0–103.5 nm and 0–106.39 nm, respectively. Several studies indicate that the heights of individual polysaccharide chains typically fall within the range of 0.1 to 1.0 nm, indicating a tendency for clustering between SC-P and SC-PF molecules [38,39].

### 2.4. The Structure and Thermal Analysis of the Polysaccharides

**Figure 3:**
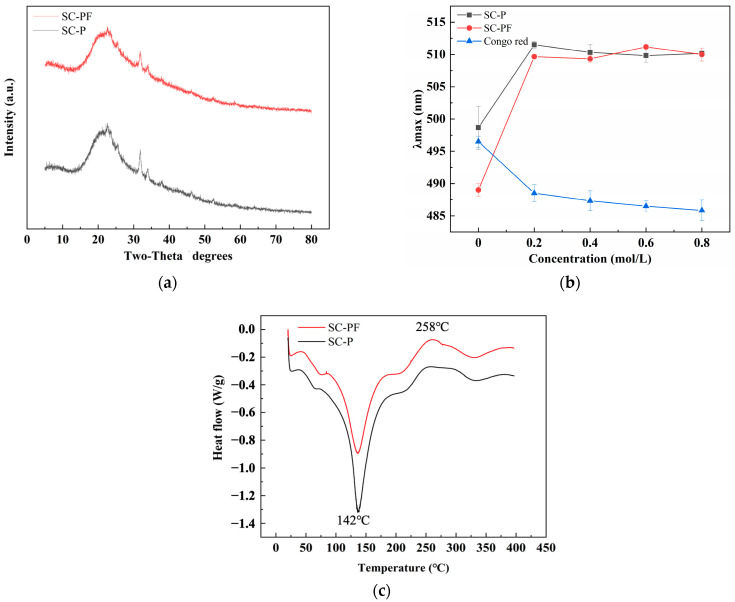
Structural and thermal analysis of the polysaccharides: (**a**) XRD analysis; (**b**) Congo Red analysis; (**c**) DSC analysis.

The crystal structure of polysaccharides is related to solubility, swelling capacity, flexibility, and viscosity [40]. The XRD spectra of SC-P and SC-PF are illustrated in Figure 3a. For SC-P and SC-PF, two sharp peaks of 2θ were observed at 31.9° and 33.9°, indicating that the polysaccharides have high crystallinity and purity. However, a curved diffraction peak of 2θ was observed at 22°, suggesting that SC-P and SC-PF are semi-crystalline substances. The present results indicate that fermentation does not have any effect on the crystal structure of the polysaccharides.

The Congo Red staining method is employed to identify triple-helical conformations in polysaccharides [41]. This structural feature is significant, as it is closely associated with the polysaccharides’ biological activities and functions. Congo Red can bind to polysaccharides with a triple helix structure, forming a complex. In a solution of a specific NaOH concentration, the complex solution’s maximum absorption wavelength shows a significant redshift compared to pure Congo Red. The formation of complexes between SC-P, SC-PF, and Congo Red is shown, with the maximum absorption wavelengths observed in the 0–0.8 M range, as depicted in Figure 3b. Under a final NaOH concentration ranging from 0 to 0.8 M, the absorbance of Congo Red complexes with SC-P and SC-PF initially increases and then levels off, while the absorbance of pure Congo Red solution shows a slow decline. Our observations confirm that both SC-P and SC-PF show a significant redshift, indicating that sea cucumber intestinal polysaccharides can bind to Congo Red, forming complexes that suggest a triple helix chain conformation for both.

The DSC spectra of SC-P and SC-PF are illustrated in Figure 3c. Within the measurement range, both SC-P and SC-PF show simultaneous endothermic and exothermic reactions as the temperature increases. This indicates that SC-P and SC-PF undergo solid configuration decomposition and transformation during heating. In the first stage of the DSC curve, a narrow endothermic peak at 142 °C is attributed to the peripheral polysaccharide chain loss, moisture evaporation, and dehydroxylation reactions. During melting, a small exothermic peak at 258 °C in the second stage is attributed to polysaccharide degradation or oxidation decomposition [42]. The DSC spectra show that SC-P and SC-PF have similar thermal characteristics.

### 2.5. The Antioxidant Characteristics of the Polysaccharides

**Figure 4:**
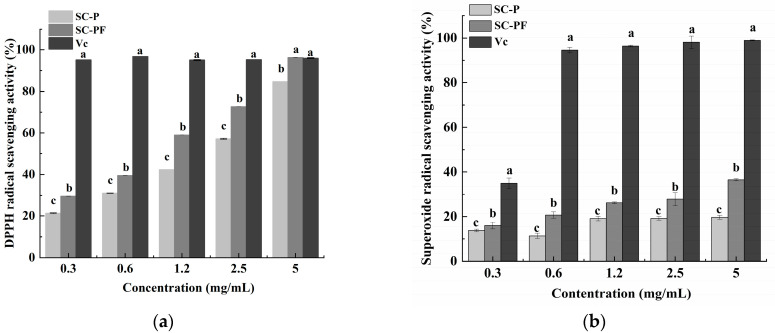
The antioxidant abilities of the polysaccharides: (**a**) DPPH scavenging ability; (**b**) superoxide radical scavenging ability. Different lowercase letters indicate statistically significant differences (*p* < 0.05).

Polysaccharides’ ability to neutralize DPPH radicals is linked to the hydrogen-donating capability of their hydroxyl groups. Polysaccharides have the capability to act as electron or hydrogen donors in the removal of hydroxyl radicals [43]. As shown in Figure 4a, between polysaccharide concentrations of 0.3 and 2.5 mg/mL, the DPPH radical scavenging activities of SC-P and SC-PF are significantly decreased compared to that of Vc at equivalent concentrations. However, at a 5.0 mg/mL concentration, the radical scavenging rates for SC-P, SC-PF, and Vc are 84.7%, 96.3%, and 96.0%, respectively. Among them, SC-PF shows the highest scavenging activity (96.3%), followed by Vc (96%) and then SC-P (84.7%). There is no significant difference in scavenging activity between SC-PF and Vc (*p* > 0.05). Within the experimental range, SC-PF shows significantly higher DPPH scavenging activity than SC-P (*p* < 0.05), which may be linked to the carboxyl or acetyl content in SC-PF. Both SC-P and SC-PF reveal concentration-dependent DPPH radical scavenging activity.

Superoxide anions serve as precursors to hydroxyl radicals and singlet oxygen. These reactive species play a role in initiating lipid peroxidation and can lead to oxidative damage, affecting proteins, DNA, and enzymes within the body [44]. The ability of polysaccharide samples to scavenge superoxide anions was assessed and compared with that of Vc. This comparison helps evaluate the effectiveness of the polysaccharides in neutralizing superoxide anions relative to a well-known antioxidant. At 5.0 mg/mL, the superoxide anion scavenging rates of SC-P, SC-PF, and Vc reached 19.7%, 36.5%, and 98.9%, respectively. Vc (98.9%) showed the highest scavenging activity, followed by SC-PF (36.5%) and then SC-P (19.7%) (Figure 4b). A positive correlation was observed between polysaccharide concentration and their scavenging effect on superoxide anions; SC-PF had significantly higher scavenging activity than SC-P (*p* < 0.05). However, the scavenging activities of both polysaccharides were markedly lower than that of Vc (*p* < 0.05). The energy required for the dissociation of the O-H bond might play a role in the mechanism of superoxide radical scavenging. Several studies have indicated that the presence of sulfate groups in polysaccharides can significantly improve their ability to scavenge superoxide radicals [2,45]. This enhancement is attributed to the increased number of electron-donating substituents on the sugar ring. This increases the electron density of the carbon atoms in the sugar ring, promotes hydrogen dissociation from the O-H bond, and stabilizes the superoxide anion.

### 2.6. Hypoglycemic and Cholesterol-Absorbing Activities

**Figure 5:**
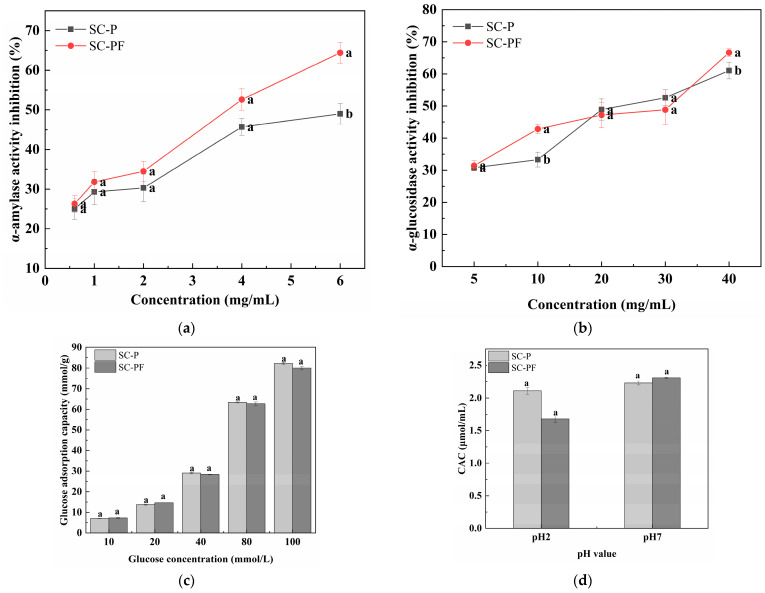
Functional properties of the polysaccharides: (**a**) α-amylase activity inhibition; (**b**) α-glucosidase activity inhibition; (**c**) Glc absorption capacity; (**d**) cholesterol absorption capacity. Different lowercase letters indicate statistically significant differences (*p* < 0.05).

The enzyme inhibitory activities of α-amylase for the two polysaccharides are shown in Figure 5a. The results indicate that from 0.5 to 4.0 mg/mL, the α-amylase inhibitory activity of the SC-PF group was slightly greater but not significantly so relative to the SC-P group (*p* > 0.05). At 6 mg/mL, the inhibitory effects of SC-P and SC-PF on α-amylase were 49.0% and 64.4%, respectively, with the SC-PF inhibition activity significantly higher than the SC-P group (*p* < 0.05). Furthermore, it was found that SC-P and SC-PF display concentration-dependent α-amylase inhibitory activity. Typically, polysaccharides inhibit α-amylase through two main mechanisms [46]. First, polysaccharides can adsorb onto starch, obstructing the enzyme’s ability to hydrolyze the starch. Second, certain functional groups in polysaccharides, such as carboxyl groups, can form hydrogen bonds with amino acid residues in α-amylase. This interaction may lead to the formation of a polysaccharide/α-amylase complex, potentially altering the spatial configuration of the enzyme and affecting its activity.

The α-glucosidase inhibition capability of polysaccharides is significant in evaluating their therapeutic potential for diabetes. The α-glucosidase inhibitory activities of two polysaccharides are shown in Figure 5b. The results indicate that at a polysaccharide concentration of 10 mg/mL, the inhibition rates of SC-P and SC-PF on α-glucosidase are 33.40% and 42.83%, respectively, with the SC-PF group showing significantly higher inhibitory activity than the SC-P group (*p* < 0.05). Furthermore, at a polysaccharide concentration of 40 mg/mL, the α-glucosidase inhibitory activities of SC-P and SC-PF are 61.03% and 66.63%, respectively, with SC-PF still showing significantly higher inhibition compared to SC-P. Both polysaccharides demonstrate concentration-dependent inhibitory activities on α-glucosidase. Previous research has demonstrated that -OH and -COOH groups on the polysaccharide side chains undergo hydrogen bonding with α-glucosidase [47]. This interaction effectively inhibits the enzyme’s activity by disrupting its normal function, thus influencing the enzymatic process. As a result, polysaccharides with higher GlcA contents and lower Mw are better able to inhibit α-glucosidase due to increased exposure to active sites [48]. The higher inhibitory activity of SC-PF on α-glucosidase may be due to its lower Mw and higher GlcA content, providing further evidence for SC-PF as a potential candidate drug for α-glucosidase inhibition.

Adsorbing Glc reduces free Glc content, thereby reducing postprandial blood Glc levels. The adsorption of Glc by SC-P and SC-PF at varying Glc concentrations (10–100 mM) is shown in Figure 5c. The GAC of the polysaccharides SC-P and SC-PF was directly linked to the Glc concentration. At higher Glc concentrations (100 mM), the GACs of SC-P and SC-PF are 82.2 and 80.0 mmol/g, respectively (Figure 5d). However, an insignificant difference was observed between the SC-P and SC-PF for the GAC and CAC.

## 3. Materials and Methods

### 3.1. Materials

The SIs were obtained from Dalian Food Jinzhou Co., Ltd. (Liaoning, China). *Enterococcus hirae* GS22 was isolated from the soil and preserved in the China General Microbiological Culture Collection Center (No. CGMCC 26984). DeMan, Rogosa, and Sharpe (MRS) broth were sourced from AOBOX Biotechnology Co., Ltd. (Beijing, China). The monosaccharide standards used, including arabinose (Ara), galacturonic acid (GalA), galactose (Gal), fucose (Fuc), glucuronic acid (GlcA), rhamnose (Rha), ribose (Rib), glucose (Glc), mannose (Man), and xylose (Xyl), were sourced from Sigma-Aldrich (St. Louis, MO, USA). Papain (800,000 U/g) was obtained from Beijing Jinming Biotechnology Co., Ltd. (Beijing, China). Shanghai Aladdin Biochemical Technology Co., Ltd. (Shanghai, China) supplied trichloroacetic acid (TCA), with all other reagents being of analytical grade.

### 3.2. SI Fermentation

SI fermentation was performed according to Li’s methods with some modifications [20]. The sea cucumber intestines were thawed at 4 °C then washed thoroughly with deionized water and cut into small pieces using scissors. The chopped sea cucumber intestines were mixed with distilled water (1:5, *w*/*v*), and papain (16,000 U/g) was added. The mixture was then enzymatically digested at 60 °C for 24 h to obtain the enzymatic hydrolysate of the sea cucumber intestines. The enzymatic hydrolysate was subsequently centrifuged (5000 rpm, 30 min) to collect the supernatant, resulting in the sea cucumber intestinal hydrolysate (M). Following this, M underwent enzyme inactivation and sterilization. After cooling to room temperature (23–25 °C), *E. hirae* GS22 was inoculated at a 6.5 log CFU/mL concentration without adding any nutritional supplements in a 500 mL flask, which was sealed with a sealed film. Fermentation was carried out at 37 °C for 48 h, followed by sterilization and centrifugation to obtain the fermented sea cucumber intestinal hydrolysate (MF). During fermentation, fermentation broth was collected at intervals of 0, 6, 12, 24, and 48 h to monitor the change in the pH value and the bacterial survival condition of the MF. pH values (PHS-25, INESA, Shanghai, China) and the growth of strains (via the plate counting method) were measured.

### 3.3. Polysaccharide Extraction

Each SI sample, with or without fermentation, was centrifuged at 3500× *g* for 15 min. An equal volume (1:1, *v*/*v*) of the supernatant was mixed with 5% TCA and incubated overnight. The mixture underwent another round of centrifugation at 3500× *g* for 15 min, after which it was combined with 95% ethanol (1:3 *v*/*v* ratio) followed by allowing it to stand for 12 h at 4 °C. Finally, it was centrifuged under the same conditions. The resulting precipitate was lyophilized to obtain the sea cucumber polysaccharides SC-P (without fermentation) and SC-PF (*E. hirae* GS22 fermentation).

### 3.4. Chemical Composition Analysis of the Polysaccharides

The carbohydrate content in the polysaccharides was assessed through the phenol-sulfuric acid method using Glc as the standard. Protein quantification was performed with Bradford assays with BSA as the standard. Reducing sugar content was identified via the DNS method, while the carbazole sulfuric acid method was employed to measure the uronic acid content, referencing GlcA [49].

### 3.5. Characterization of Polysaccharide Molecular Mass Distribution and Monosaccharide Composition

The SI polysaccharides’ average Mw was identified using high-performance gel permeation chromatography (HPGPC) (Waters1525, Dalian Elite Analytical Instruments Co., Ltd., Dalian, China). The analysis used a PL aqua gel-OH MIXED 8 μm column (Agilent Technologies, Santa Clara, CA, USA) [50]. Various Mws of dextran (6.7 × 10^5^, 4.1 × 10^5^, 2.7 × 10^5^, 5 × 10^4^, 2.5 × 10^4^, 1.2 × 10^4^, 5 × 10^3^, and 1 × 10^3^ Da) were utilized as standards. The HPGPC software program was used to calibrate the standard curve.

To evaluate the monosaccharide composition of the polysaccharides, high-performance liquid chromatography (HPLC) was employed using an Agilent 1260 system. The protocol was followed as previously described with some modifications [51]. A hydrolysis reaction was conducted on 5 mg polysaccharide samples in a 2 M trifluoroacetic acid (TFA) solution for the duration of 6 h at 100 °C. The TFA was eliminated by co-evaporating it with methanol. After the solution was concentrated under vacuum to dryness, the resulting hydrolysis product was reconstituted in 100 μL of 0.3 M NaOH. This alkaline solution was then reacted with 0.5 M 1-phenyl-3-methyl-5-pyrazolone (PMP) for 1 h at 70 °C, after which it was combined with 100 μL of 0.3 M HCl and centrifuged (2400× *g*, 5 min). After filtration (0.22 μm membrane), the supernatant was injected into an Xtimate C18 column (4.6 × 200 mm, 5 μm) on a Shimadzu LC-20AD (Kyoto, Japan) for HPLC analysis. The analysis used a mobile phase of 0.1 M KH_2_PO_4_ (pH 10) and acetonitrile in an 83:17 ratio, with a flow rate of 1.0 mL/min and the column temperature set at 30 °C.

### 3.6. Particle Size and Zeta Potential Profiling

The average particle size of the polysaccharides was evaluated using dynamic light scattering (DLS) with a Nano-ZS90 instrument (Malvern, UK), while the zeta potential was assessed with a 90 Plus Zeta instrument (Brookhaven Instrument Corporation, Nashua, NH, USA) [52].

### 3.7. Spectral and Morphology Analysis

#### 3.7.1. Spectral Analysis

The UV spectra between 200 and 800 nm were assessed with a Shimadzu UV-2550 spectrophotometer. Fourier-transform infrared spectroscopy (FT-IR) was conducted by a Scimitar 2000 FT-IR spectrometer (Agilent) covering a wavelength from 400 to 4000 cm^−1^. This setup allows for a comprehensive analysis of the samples, providing detailed insights into both the UV-absorbing characteristics and the infrared spectral properties [53].

#### 3.7.2. Scanning Electron Microscopy (SEM) Analysis

Lyophilized polysaccharides were affixed to a copper platform with a conductive adhesive, followed by treatment with gold spray. The morphology of the SI polysaccharides was tested through SEM (S-4800, Hitachi Ltd., Tokyo, Japan) at a 10 kV acceleration voltage with magnifications of 200×, 500×, 2000×, and 5000× [54].

#### 3.7.3. Atomic Force Microscopy (AFM) Analysis

A 5 μg/mL solution of the sample was placed on a mica surface (freshly cleaved) and allowed to dry with nitrogen gas under room temperature and then observed using an AFM (SPI3800-SPA-400; NSK Ltd., Tokyo, Japan).

### 3.8. Structural and Thermal Characteristic Analysis

#### 3.8.1. X-Ray Diffraction (XRD) Analysis

The XRD of the polysaccharides was measured with a D8 diffractometer (Bruker AXS, Karlsruhe, Germany) between 20° and 80° with an applied voltage of 30 kV and a current of 30 mA [55].

#### 3.8.2. Congo Red Analysis

For the Congo Red test, a polysaccharide solution (1 mL, 2.0 mg/mL) was mixed with 1.5 mL of a 0.2 mM Congo Red solution. The control was distilled water only. The resulting mixture was mixed sequentially with NaOH solutions (0.1, 0.2, 0.3, 0.4, 0.6, and 0.8 M). Then, it was mixed with 0.5 mL distilled water and agitated vigorously. The sample was then scanned to determine the peak absorption wavelength.

#### 3.8.3. Differential Scanning Calorimetric (DSC) Analysis

The polysaccharides’ thermal properties were evaluated using a DSC 8000 Calorimeter from PerkinElmer (Shelton, Georgia, GA, USA) [56]. Samples weighing 5 and 10 mg were carefully placed into an aluminum crucible and then sealed with an aluminum lid. An empty crucible was used as the blank control. The thermal analysis measured the heat flux intensity over temperature increases from 20 to 400 °C at 10 °C/min.

### 3.9. Functional Analysis

#### 3.9.1. Determination of Antioxidant Activity

The DPPH scavenging ability of the polysaccharide was evaluated following the procedure outlined by Zhou et al. [57]. Scavenging activity was determined by Equation (1), where A_t0_ and A_t30_ represent the OD taken at 517 nm before and 30 min after the reaction, respectively.

(1) $$\mathrm{DPPH}\;\mathrm{inhibition}\;(\%)=\frac{{(A}_{t0}-A_{t30})}{A_{t0}}\times 100$$

To assess the scavenging rate of superoxide anion radicals, 1 mL of EPS solutions (0.3 to 5 mg/mL) was added to 0.3 mL of a 3 mM gallic acid solution. This mixture was then added to 4.5 mL of Tris-HCl buffer (50 mM, pH 8.2), shaken well, and kept for 5 min at 25 °C in darkness. The reaction was terminated with 10 mM HCl, after which the OD at 320 nm was recorded. Ascorbic acid (Vc) represented the positive control, and the rate at which it scavenged the superoxide anion radical was determined as Equation (2) [58].

(2) $$\mathrm{Superoxide}\;\mathrm{anion}\;\mathrm{radical}\;\mathrm{scavenging}\;\mathrm{rate}\;\left(\%\right)=\left[1-\;\left(A_{1}-A_{2}\right)/A_{3}\right]\times 100$$

A_1_ is the OD of the sample, and A_2_ is the OD of the mixture of the sample solution without the pyrogallol solution and PBS. A_3_ is the OD of the control.

#### 3.9.2. Inhibition Capacities of α-Amylase

Next, 500 μL of samples of varying concentrations was mixed with 500 μL of the α-amylase solution, which was prepared at a concentration of 1 U/mL in a 0.1 M phosphate buffer at pH 6.8 and pre-incubated at 37 °C for a duration of 10 min to ensure proper interaction between the polysaccharides and the enzyme. This pre-incubation step is crucial for allowing the polysaccharides to interact with the α-amylase, setting the stage for successive enzymatic activity assays. Following this, a 500 μL starch solution (1%, *w*/*v*) was introduced into the mixture, followed by subjecting it to a 10 min incubation at 37 °C. The reaction was stopped by heating at 100 °C for 5 min and adding 1 mL of a 3,5-dinitrosalicylic acid (DNS) solution. After the mixture was allowed to cool, 10 mL of DI water was added. The final concentrations of the polysaccharides in the solution were 0.12, 0.2, 0.4, 0.8, and 1.2 mg/mL. Acarbose was used as a positive control for comparison. The optical density at 540 nm (OD_540_) was read, and the rate of inhibition was determined using Equation (3) [59].

(3) $$\alpha \text{-}\mathrm{amylase}\;\mathrm{activity}\;(\%)=\left(1-\frac{A_{3}-A_{4}}{A_{1}-A_{2}}\right)\times 100$$

where *A*_3_ represents the absorbance of the mixture, *A*_1_ is the absorbance of the solution that does not contain inhibitors, *A*_2_ is the absorbance of the solution that does not contain enzymes and inhibitors, and *A*_4_ is the absorbance of the solution that does not contain enzymes.

#### 3.9.3. Inhibition Capacities of α-Glucosidase

To determine the α-glucosidase inhibition effects of the polysaccharides, all reagents were prepared in PBS (pH 6.9, 0.1 M). Briefly, the mixture was prepared by combining 40 μL of the polysaccharides at different concentrations with an equal volume of 40 μL of α-glucosidase and then incubating it at 37 °C for 15 min. Following the initial incubation, 20 μL of a 16 mM solution of 4-nitrophenyl-α-d-glucopyranoside (PNPG) was added to the mixture, which was then incubated at 37 °C for an additional 15 min. After this, 150 μL of 0.2 M sodium carbonate (Na_2_CO_3_) was used to stop the reaction. Different concentrations of acarbose (1.6, 8, 16, 80, and 160 μg/mL) were used as positive controls. The OD of the resulting mixture was read at 405 nm (A), and the inhibition rate of α-glucosidase was determined using Equation (4) [59].

(4) $$\alpha \text{-}\mathrm{glucosidase}\;\mathrm{inhibition}\;\mathrm{rate}\;(\%)=\left(1-\frac{A_{S}-A_{b}}{A_{b}}\right)\times 100$$

where *A_s_* is the OD of the mixture, *A_b_* is the OD without α-glucosidase, and *A_b_* is the absorbance without polysaccharides. The missing polysaccharides and enzymes were replaced with an equal amount of PBS.

#### 3.9.4. Glucose Absorption Capacity (GAC)

Different SI polysaccharides (0.025 g each) were dissolved in 25 mL of Glc at varying concentrations (10, 20, 40, 80, and 100 mM), incubated in a water bath at 37 °C for 6 h, and centrifuged (3500× *g*, 15 min), after which the Glc content in the supernatant was measured by the phenol-sulfuric acid method. For the positive control, guar gum was used, and the GAC was derived from Equation (5) [60].

(5) $$\mathrm{GAC}\;(\mathrm{mmol}/g)=\frac{G1-G2}{W}\times V$$

where G1 represents the initial concentration of Glc, G2 represents the concentration of Glc after centrifugation, W is the mass of the polysaccharide, and V is the volume of the mixture.

#### 3.9.5. Cholesterol Absorption Capacity (CAC)

Egg yolk and DI water were mixed in a 1:9 (*v*/*v*) ratio and homogenized to produce a uniform emulsion. The dried polysaccharide sample was combined with the prepared emulsion at a 1:5 (*w*/*v*) ratio, with adjustment of the pH to 2.0 to mimic the conditions of human gastric fluid or to 7.0 to simulate the pH of the jejunum. The solutions were then incubated in a shaking water bath for 120 min at 37 °C and then centrifuged (4000× *g*, 15 min, 25 °C). The cholesterol content was estimated using a test kit, and the amount of cholesterol adsorption was assessed using Equation (6) [60].

(6) $$\mathrm{CAC}\;\left(\mu \mathrm{mol}/\mathrm{mL}\right)=C_{1}-C_{2}$$

C_1_ is the amount of cholesterol in the egg yolk (in μmol/mL), and C_2_ is the amount in the supernatant (in μmol/mL).

### 3.10. Statistical Analysis

All experiments were conducted in triplicate to ensure the reliability and reproducibility of the results. Data were analyzed under the assumption of a normal distribution, and variance analysis was performed to assess the differences between groups. In order to further examine specific group differences, Tukey’s post hoc test was employed for pairwise comparisons of means. All statistical analyses were conducted using SPSS version 18.0; *p*-values < 0.05 were considered statistically significant.

## 4. Conclusions

The present results indicated that *E. hirae* GS22 fermentation could influence the extraction yield, Mw, monosaccharide composition, microstructure, and prebiotic activities of the SI polysaccharides. After fermentation, the yield of the SI polysaccharides significantly increased from 0.48% to 0.63%, with SC-PF showing substantially higher antioxidant activity, α-amylase inhibition, and α-glucosidase inhibition activity than SC-P. It is proven that *E. hirae* GS22 fermentation would be an effective method to modify SI polysaccharides and improve their biological function. It is also a promising technique that could simulate and substitute the enzyme hydrolysis current commonly used in the food processing industry. Future studies should pay more attention to the mechanism of the functional improvement of fermentation on the SI polysaccharides and verify their metabolite pathways in animal models.

## Figures and Tables

**Table 1:** Chemical properties of the polysaccharides.

Item	SC-P	SC-PF
Yield (%)	0.48 ± 0.06 ^b^	0.63 ± 0.06 ^a^
Carbohydrate (%)	44.40 ± 0.10 ^b^	50.35 ± 0.01 ^a^
Protein (%)	3.30 ± 0.00 ^a^	3.98 ± 0.20 ^a^
Reducing sugar (%)	3.65 ± 0.34 ^a^	5.10 ± 0.63 ^a^
Uronic acid (%)	0.23 ± 0.00 ^b^	0.29 ± 0.01 ^a^
Molecular weight distribution (Da)	35,964	32,022
Monosaccharide composition (molar ratio, %)		
Mannose (Man)	2.96	5.55
Ribose (Rib)	7.80	10.07
Glucuronic acid (GlcA)	0.10	1.17
Galacturonic acid (GalA)	0.02	0.06
Glucose (Glc)	7.63	13.4
Galactose (Gal)	1.49	3.12
Fructose (Fuc)	1.26	1.72
Xylose (Xyl)	-	0.04
Arabinose (Ara)	0.19	0.37
Particle size (nm)	2005.09 ± 82.73 ^a^	1285.77 ± 168.85 ^b^
Polydispersity index	0.337 ± 0.013 ^a^	0.362 ± 0.023 ^a^
Zeta potential (mv)	−16.49 ± 0.53 ^a^	−21.42 ± 1.38 ^b^

Note: Different letters indicate the presence of significant differences between samples (*p* < 0.05). - means not detected.

## Data Availability

Data are contained in the article.
